# Music Therapy Interventions for Stress Reduction in Adults With Mild Intellectual Disabilities: Perspectives From Clinical Practice

**DOI:** 10.3389/fpsyg.2020.572549

**Published:** 2020-12-10

**Authors:** Martina de Witte, Esther Lindelauf, Xavier Moonen, Geert-Jan Stams, Susan van Hooren

**Affiliations:** ^1^Research Institute of Child Development and Education, University of Amsterdam, Amsterdam, Netherlands; ^2^Academy of Health and Vitality, HAN University of Applied Sciences, Nijmegen, Netherlands; ^3^Stevig, Expert Centre for People with Mild Intellectual Disabilities, Gennep, Netherlands; ^4^KenVaK, Research Centre for the Arts Therapies, Heerlen, Netherlands; ^5^Faculty of Healthcare, Zuyd University of Applied Sciences, Heerlen, Netherlands; ^6^Faculty of Psychology and Educational Sciences, Open University, Heerlen, Netherlands

**Keywords:** relaxation, mild intellectual disabilities, stress release, stress, music therapy, improvisation (music), mechanisms of change, change factors

## Abstract

Stress is increasingly being recognized as one of the main factors that is negatively affecting our health, and therefore there is a need to regulate daily stress and prevent long-term stress. This need seems particularly important for adults with mild intellectual disabilities (MID) who have been shown to have more difficulties coping with stress than adults without intellectual disabilities. Hence, the development of music therapy interventions for stress reduction, particularly within populations where needs may be greater, is becoming increasingly important. In order to gain more insight into the practice-based knowledge on *how* music therapists lower stress levels of their patients with MID during music therapy sessions, we conducted focus group interviews with music therapists working with adults with MID (*N* = 13) from different countries and clinical institutions in Europe. Results provide an overview of the most-used interventions for stress reduction *within* and *outside* of music. Data-analysis resulted in the further specification of therapeutic goals, intervention techniques, the use of musical instruments, and related therapeutic change factors. The main findings indicate that music therapists used little to no receptive (e.g., music listening) interventions for stress reduction, but preferred to use active interventions, which were mainly based on musical improvisation. Results show that three therapy goals for stress relief could be distinguished. The goal of “synchronizing” can be seen as a sub goal because it often precedes working on the other two goals of “tension release” or “direct relaxation,” which can also be seen as two ways of reaching stress reduction in adults with MID through music therapy interventions. Furthermore, the tempo and the dynamics of the music are considered as the most important musical components to reduce stress in adults with MID. Practical implications for stress-reducing music therapy interventions for adults with MID are discussed as well as recommendations for future research.

## Introduction

Stress reduction has become increasingly important in health care practices of many professional fields, including music therapy. Continuous high levels of stress have been shown to negatively affect our health (e.g., Australian Psychological Society [APS], 2015; American Psychological Association [APA], 2017). Although many people have found ways to adequately cope with stress and its possible negative consequences, it still appears to be difficult for a substantial number of people to cope with daily stressors, and the demands of contemporary life (Holahan et al., 2005; World Health Organization [WHO], 2010; Casey, 2017; De Witte et al., 2020b). This especially may be the case for adults with mild intellectual disabilities (MID^1^), who have been shown to experience much more stress in daily life than people without intellectual disabilities (Emerson, 2003; Hatton and Emerson, 2004; Schuengel and Janssen, 2006), having fewer resources to cope with daily life stress (Lunsky and Benson, 2001; Hartley and MacLean, 2009b; Scott and Havercamp, 2014). Therefore, it is important to gain more insight into the effectiveness and applicability of therapeutic interventions for stress reduction for adults with MID.

Music therapy is often applied to reduce stress for adults with MID (Moonen and Didden, 2014; De Witte et al., 2020a,b). The experience-based approach in music therapy is believed to be well suited to the needs of adults with MID. Although more and more studies are published on the *effects* of music therapy on stress-related outcomes (see for an overview: De Witte et al., 2020a), more information is needed regarding specific techniques, instruments, supposed change factors, requisites, and procedures within these interventions.

### Music Therapy

Music therapy is defined as the clinical and evidence-informed use of music interventions^2^ to accomplish individualized goals within a therapeutic relationship in order to meet physical, emotional, mental, social and cognitive needs (American Music Therapy Association [AMTA], 2018; De Witte et al., 2020a; Agres et al., under revision). In contrast to health care staff providing prerecorded music listening interventions (often referred to as music medicine), music therapy interventions are offered by a qualified music therapist in relation to assessment and treatment planning. Both music medicine as well as music therapy can be useful and important. According to the music therapy literature, music therapy interventions can be divided in two broad categories: active and receptive interventions (Bruscia, 2014; Wheeler, 2015; Magee, 2019).

Active interventions involve the patient making music during the music therapy session, while receptive interventions mean that the patient is only receiving the music, such as listening to live or prerecorded music. Whereas “music medicine” does not involve a therapeutic process, music therapy requires the presence of such a process through personally tailored music experiences, including listening to music (Grocke and Wigram, 2006; Bruscia, 2014; Bradt et al., 2015). Bruscia (2014) described a more refined classification of music therapy interventions/methods, resulting in four categories: improvisation, recreating (or performing), and composing, which are active interventions, and music listening, which refers to receptive interventions.

Music therapists are specifically trained to use the unique qualities of music, also known as musical components (e.g., melody, rhythm, tempo, dynamics, pitch) within the therapeutic relationship to work on patient’s treatment goals (Bruscia, 1987; Wheeler, 2015; De Witte et al., 2020a). During the music therapy session, the music therapist attunes to the patient by adjusting the way of music-making as an immediate response to the patient’s needs (Aalbers et al., 2019; Magee, 2019). In music therapy literature, this way of patient-therapist attunement is often related to the term “synchronization,” which means that the music therapist and the patient interact simultaneously and are regulated through time, yielding a similar expression in movement, matching pulse, rhythm, dynamics and/or melody (Bruscia, 1987; Schumacher and Calvet, 2008; Aalbers et al., 2019; De Witte et al., 2020a). More specifically, synchronization in music therapy may refer to both the way music therapists intervene within different kinds of interventions, and to a specific music therapy technique for musical improvisation developed by Bruscia (1987), which can help music therapists to resonate emotions and/or expressions in the music or resonate non-verbal behavior. In this study, we consider synchronization in a broader sense.

In addition, music therapists often perform interventions based on rich, experiential, tacit knowledge. Tacit knowledge refers to practice-based knowledge developed from the direct experiences within clinical practice, which is mainly subconsciously understood and applied (McAdam et al., 2007; Polanyi, 2009; Petri et al., 2020). Using tacit knowledge is common in professionals in clinical practice and guides the professional in treating patients based on professionals’ previous experiences. However, making tacit knowledge explicit may stimulate transferability of valuable clinical practices, and may stimulate a different way of thinking about the relationship between clinical practice, theory, and research (Aigen, 1999; Smeijsters and Vink, 2006; Stige, 2015); furthermore, making tacit knowledge explicit may lead to the development of intervention descriptions derived directly from clinical practice, such as intervention manuals or therapy protocols, for which there is still a great need (Aalbers et al., 2019). From the scientific point of view, clear intervention descriptions are needed to further investigate what exactly is effective in music therapy interventions or to replicate outcome studies (Hoffmann et al., 2014). Our previous meta-analysis on the effects of music therapy on stress reduction showed, for example, that 63% of the included studies provided important details of the music therapy interventions examined, but only 35% of the studies reported on the use of therapy protocols or manuals (De Witte et al., 2020a). Therefore, it is important for both clinical practice and future research to systematically illuminate the knowledge already present in clinical practice, such as the tacit knowledge of music therapists.

### Experience of Stress in People With MID

Stress can be defined as the quality of an experience, produced through a person-environment transaction that, through either overarousal or underarousal, results in psychological and/or physiological distress (Aldwin, 2007; Riley and Park, 2015). Adults with MID, also defined as a neurodevelopmental disability characterized by deficits in intellectual and adaptive functioning skills, may demonstrate difficulties in coping with stress (American Psychiatric Association [APA], 2013). As in the general population, (continuous) high levels of stress experienced by adults with MID is associated with many negative mental health outcomes (Hulbert-Williams and Hastings, 2008; Hartley and Maclean, 2009a; Hartley and MacLean, 2009b; Scott and Havercamp, 2014).

In general, social support and self-efficacy can be considered as important factors for stress resistance (Everly and Lating, 2019). These factors are less developed in adults with MID (Abbaszadeh and Sardoie, 2016; Seyed et al., 2017). The relatively high impact of daily stressors, such as taxing social interactions, may also be explained by the lack of control over both minor daily and major life decisions (Hartley and MacLean, 2009b; Dulin et al., 2013; Scott and Havercamp, 2014). When stress experiences continue in adults with MID, it can lead to an increase of maladaptive coping strategies and serious health issues, such as depression (Hartley and Maclean, 2009a; Hartley and MacLean, 2009b), impaired cognitive functions (Heyman and Hauser-Cram, 2015), physical health problems (Lunsky, 2008), and substance abuse (Didden et al., 2009).

In clinical practice, the content of psychosocial approaches to interventions for adults with MID varies from strict cognitive-behavioral to that which is more experiential. Experiential approaches refer to those instances where the patient can learn through action-based experiences. Experiential approaches to intervention may allow for adults with MID to more readily address the reduction of stress. Experiential approaches to intervention focus on the “here and now” while guided by a therapist through stress responses and real-time emotional regulation. Through safely structured active experiences, stress inducing situations can be co-navigated, and stress reducing strategies can be developed and/or practiced (De Witte et al., 2020a, 2017).

The efficacy of action-based experiences in interventions for adults with MID has been demonstrated in a study on the effects of the different components of cognitive-behavioral interventions for adults with MID. Studies have shown that behavioral activation strategies alone, such as roleplay exercises, resulted in the same outcome as the full cognitive therapy package (Cuijpers et al., 2007; Hamelin et al., 2013; Didden et al., 2016). The cognitive-behavioral approach often results in patients being able to recognize that their thinking is not logical, but they do not feel emotionally different afterward (Patterson et al., 2019). This lack of emotional address suggests the need for the further development of interventions based on experiential approaches to intervention, including those that occur within music therapy processes.

### Music Therapy for Adults With MID

Despite the lack of clinical effectiveness studies on music therapy for adults with MID, clinical practice shows that music therapists can meet the needs of adults with MID very well (Watson, 2002; Hooper et al., 2011; Hoyle and McKinney, 2015). A scoping review (Hooper et al., 2008a,b) provides a comprehensive summary of published studies about music interventions/activities for adults with learning and/or intellectual disabilities documented from 1943 to 2006; the researchers primarily located practice-based evidence from small sized studies of music interventions. Studies that specifically focused on music therapy with adults with MID were lacking. The majority of the published studies involved children with intellectual disabilities in a school setting (Meila, 2017), or concerned case study reports of music therapy processes in which stress regulation was not the targeted therapeutic goal (e.g., Brennand et al., 2011; Hoyle and McKinney, 2015).

Since 2006, no studies have been conducted in which the content of music therapy interventions aimed at stress reduction for adults with MID was described clearly and systematically. However, there is a growing body of neuropsychological evidence showing the positive influence of music on lowering people’s stress levels (Chanda and Levitin, 2013; Thaut and Hoemberg, 2014; Koelsch et al., 2016). Two meta-analytic studies showed positive overall effects of music listening interventions on stress reduction in different kinds of settings (Pelletier, 2004; De Witte et al., 2020b). A recent meta-analytic review of 47 studies shows medium-to-strong effects (*d* = 0.723) of specifically *music therapy* on psychological and physiological stress-related outcomes (De Witte et al., 2020a).

### Purpose of the Present Study

The purpose of this study is to systematically collect practice-based knowledge on both the most efficient music therapy interventions, such as therapeutic methods or exercises, and elements of those interventions, such as musical *techniques*, used by music therapists to reduce stress in patients with MID. This information may contribute to the body of knowledge on *how* music therapists lower the stress levels of their patients with MID during music therapy sessions. To be more specific, we wanted to know which interventions both within and outside of the music are used by the music therapists to lower the patients’ stress levels, and which factors may influence the choice of the applied intervention(s).

## Materials and Methods

To collect this practice-based knowledge, we set up multiple focus groups (Litosseliti, 2003; Liamputtong, 2011; Kitzinger, 2013) with music therapists from three different countries and different types of clinical institutions who practice music therapy with adults with MID. Focus group methodology is characterized by guided group discussions to generate a rich understanding of participants’ experiences and their tacit knowledge (Morgan, 1997; van Bruggen-Rufi et al., 2018). A focus group discussion is aimed at eliciting perceptions, attitudes, and ideas from participants about specific topics (Vaughn et al., 1996), and to enable them to react and build on the responses of others in the group about the topics that were broached (Morgan and Scannell, 1998; Litosseliti, 2003; Liamputtong, 2011; Kitzinger, 2013).

### Participants

A total of 13 music therapists (male *n* = 6, female *n* = 7) based in Netherlands (*n* = *7*), Belgium (*n* = 3), and Germany (*n* = 3), who practice music therapy in institutions exclusively providing clinical treatment to adults with MID took part in three focus group interviews. Two music therapists were working in the same clinical institution, but in different departments. The age of the participating music therapists varied from 25 to 63 years, and overall, they were trained at different universities in Netherlands, Belgium, and Germany. We chose to include music therapists from different countries, as results related to the specific context of one country may not be automatically generalizable to other countries. The study was approved by the Ethical Research Committee of the HAN University of Applied Sciences in Netherlands (ref. no: 198.09/20).

### Procedure

Purposive sampling was applied during the recruitment process. Purposive sampling allows for the selection of participants based on specific study driven variables or characteristics (Patton, 2014; Valerio et al., 2016). This sampling strategy was considered appropriate to ensure a group of participants that would be representative of the overall population of music therapists (Dörnyei, 2007; Etikan et al., 2016). Therefore, participants were recruited from a variety of clinical institutions, taking into consideration age, gender, country of origin, and educational background. The intention for diversity sought to ensure that overall findings would not be influenced by over-representation in these areas. Once selected, music therapists were invited by email to participate in this focus group study. The music therapists who asked for more information received a second email in which the general theme of the study was described. Since the aim of the present study is to gather practice-based knowledge, no in-depth information or topic related literature was shared prior to the focus group meetings. Before the focus group meetings started, the participants were asked to sign an informed consent form, in which they clarified their willingness to participate, and their spoken data could be used anonymously for the purpose of this study.

Eligible focus group participants were (a) qualified and board-certified music therapists (b) who apply music therapy to patients with MID, (c) within a clinical or healthcare setting in which (d) patients were specifically referred to music therapy by a psychologist or psychiatrist. Music therapists without significant experience with patients with MID, such as those who had just recently started working as music therapists or those who have only treated a small number of patients with MID, were not approached. Furthermore, we did not include music therapists working in a private practice, because in this setting it is not always clear whether a patient meets the diagnostic criteria of MIDs.

### Data Collection

Prior to the focus group interviews, an interview guide was developed in which the research question, the purpose of the study, the topics and preconditions of the focus group were set up. Subsequently, a time-bound questioning route was developed, in which questions and sub-questions were formulated regarding each topic. In order to stimulate a rich discussion about which interventions music therapists use to reduce stress in adults with MID, we included questions about both interventions *within* the music, as well as interventions *outside* the music, and possible effect moderating conditions (see Supplementary Appendix A for a more extensive description of the questioning route). Although the questioning route was followed to keep the discussion on track without inhibiting the flow of ideas, the focus group promoted an open character by stimulating all participants to give their views on the subject. For example, by using follow up questions, researchers could give participants the space to respond to each other’s views.

The focus group was led by a moderator with advanced research skills and a Master’s degree in music therapy. An assistant moderator was also present to ensure that the equipment for the audio recordings worked correctly, to manage the time and take notes during the focus groups, and to join the conversation if warranted. A general introduction of the research project was given, before starting the focus group discussion. After each focus group session, the moderator and the assistant moderator evaluated the notes taken. The first focus group session included the participants who were working in Netherlands. Subsequently, two identical focus group sessions were held, one with Belgium-based participants and one with participants based in Germany.

### Data Analysis

After data-saturation was established, researchers analyzed the information using the coding principles of qualitative content analysis. Qualitative content analysis is frequently applied to answer questions such as *what*, *why*, and *how*, whereby the common patterns in the data are deduced by using a consistent set of codes to organize text into identified categories of similar meanings (Nandy and Sarvela, 1997; Moretti et al., 2011; Cho and Lee, 2014). Qualitative content analysis is based on naturalistic inquiry, which entails identifying themes and patterns, and involves rigorous coding (Moretti et al., 2011; Cho and Lee, 2014).

In preparation of the coding process, the audio recordings of the focus group sessions were fully transcribed. Then, consistent with the principles of content analysis, we applied three successive coding steps (i.e., open, axial, and selective coding) to analyze the transcripts (Corbin and Strauss, 2008). The first open-coding step was conducted by two researchers, who independently coded relevant text fragments based on “*in vivo* codes.” The relevant text fragments were labeled with the literal terms used by the participants (Corbin and Strauss, 2008). In case of disagreement between the two researchers, the topics were discussed by a larger group of researchers (the co-authors of this article) and resolved by consensus. The second step involved axial coding, where the open codes were grouped into categories based on their more overarching similarities to the property and dimension levels (Corbin and Strauss, 2008). During this axial-coding process, it became possible to define preliminary analytical (sub)categories and compare these. The third step concerned selective coding, in which the categories formed during the axial coding procedure were connected in order to create and refine an integrating model (Charmaz, 2003).

The cycle of data-analysis (the three coding steps: open, axial and selective coding) was executed by two different researchers in which an iterative process and “constant comparison” were the leading principles (Corbin and Strauss, 2008). To ensure that the findings were grounded in the initial data, every step of the data-analysis was continuously audited/coached by both a professor specialized in music therapy and a professor specialized in mental health issues of adults with MID.

### Criteria of Trustworthiness

To meet the criteria for trustworthiness, several techniques were applied to enhance the quality of the present focus group study (e.g., Creswell, 1998; Denzin and Lincoln, 2005). Transferability was strengthened by the fact that the included music therapists were employed in a variety of mental health care centers that specialize in the treatment of patients with MID.

To minimize possible bias of the interviewer, member checking took place, which met the criteria for credibility. In preparation of the analysis, the co-moderator summarized the content of each focus group discussion, which subsequently was member-checked by the participants individually by email. All the participants agreed with this report, and none of them suggested any additional comments or changes. The fact that every step of data-analysis was continuously audited by other research experts, helped to ensure that the findings were not biased by the main investigator’s motivation or interest, thus meeting the criteria of confirmability.

## Results

The open coding initially showed a mixture of many different types of music therapy interventions, in which a distinction could be made between interventions within the music and interventions outside of the music.

### Therapy Goals for Stress Reduction

Three types of therapeutic goals were mentioned by the music therapists related to stress reduction in adults with MID: synchronizing with the patient (as a starting point for stress reduction), releasing stress or tension (by self-expression), and stimulating relaxation. The music therapists stated that in general these goals do not stand alone but are used in succession of or in combination with each other. This means that the goal of achieving synchronization with the patient within the music can be seen as a sub goal, and often precedes working on the goals related to the release of tension or direct relaxation.

“I think when treating patients with MID, they first have to come into their normal tension level before start working on other treatment goals. So, every single music therapy session is starting with the lowering of the patient’s tension level and after that we continue with the common therapy activities [participant 4, music therapist in Netherlands].”

Table 1 shows a detailed overview of all the mentioned interventions and related characteristics. Because the data did not provide a detailed description of every mentioned intervention, not all columns could be filled for all the interventions mentioned. Nevertheless, in order to provide a realistic representation of the results we chose to show all the findings, even if this meant that some areas of the table would be left blank.

**TABLE 1:** Overview of interventions.

Music therapy interventions within the music			Goal	Instruments	Specification of the intervention	Assumed change factors promoting stress reduction
Active interventions	Free improvisation		Synchronizing with the patient (as a starting point for relaxation or releasing tension)		• Attuning to the patient’s tempo • Following the patient’s dynamic • Using the same pulse	
			
			Relaxation	Piano Monochord Guitar Percussion – Drum kit – Rainmaker – Ocean drum Singing bowls Wind chimes	• Decreasing music tempo • Playing simple harmonies or chord progressions (third, fifth, octave) • Using a low tonal register • Playing a simple chord progression on piano • Inducing playfulness • Working toward musical form • Incorporating patterns • Tapping/moving along to the music • Structuring the music expression • Playing in a slow tempo • Inducing minimal changes in tempo • Inducing minimal changes in melodic intonation • Using specific Bruscia techniques: ∘ Pacing ∘ Rhythmic grounding ∘ Tonal centering	Simple instruments and harmonies may impart support, the use of complicated instruments may cause stress Triggering and synchronizing of multiple senses Music may support the sense of containment. Percussion (drum kit) may impart delimitation and physical distance from the therapist
			
			Releasing tension	Piano Drums	• Accelerating tempo • Alternating between increasing and decreasing of certain musical components • Gradually increasing musical tension • Building up dynamics • Increasing the musical tension • Collectively playing an unstructured drumroll	Shared experience by playing the piano together After an increase of tension, a decrease may be experienced more intense or more vivid
	
	Structured improvisation	Freestyling	Releasing tension	Singing and/or rap	• Playing an aggressive tight beat • Playing a heavy bass	Physically experiencing the beat, may lead to become aware of body signals and tension Freestyling may offer space for the direct expression of frustration
		
		6/8-m Improvisation	Relaxation	Drums	• Using a steady pulse of 60 bpm • Using 6/8 m • Playing the same for the duration of 5–10-min • Initiating no increases in tempo	6/8 m is soothing and relaxing, and can elicit related emotions 6/8 m can be experienced as a mantra (meditative) 6/8 m consists of a clear pulse/rhythm, which is associated with calmness
		
		Interplay on piano		Piano	• Harmonic basis offered by the therapist • Dictating patient’s tempo and pulse • Letting the patient choose minor or major scale • Instructing the patient to start with single key and later to extend play with additional keys	Experiencing intrinsic rhythm
		
		Taking turns				
		Expressing feelings in music		Instrument of own choice Symbal (as a stop signal)		
		
		Singing mantras		Singing with the use of singing bowls		The repetitive nature of mantra singing
		
		Playing intrinsic tempo		Drums	• Playing patient’s own intrinsic tempo • Playing each other’s intrinsic tempo	Experiencing your own intrinsic tempo
	
	Singing and/or playing existing songs	Greek sirtaki	Externalizing patients: Releasing tension Internalizing patients: Promoting self-expression	Therapist plays accordion Patients play a variety of percussion instruments	• Starting at a low tempo • Increasing music tempo • Shouting: 1234 HOPPA • Playing rhythm collectively • Implementing changes in tempo	Structure may promote stress release for externalizing patients Coaxing may promote stress release for internalizing patients
		
		Singing/ playing well-known songs			• Making variations of well-known songs • Starting with children’s songs • The use of age-appropriate songs	Familiarity of well-known songs
		
		Using the same opening song each session				Repetition may create sense of safety
	
	Songwriting and/or composing	Recording self-composed songs (and taking home)	Releasing tension transferred to outside the music therapy session			The use of self-composed stress relieving songs outside of the music therapy sessions (transfer)
		
		Songwriting	Releasing tension			Writing songs about stressful events, may offer exposure

Receptive interventions	Listening to pre-recorded music	Recording and/or curating personal relaxation music	Relaxation outside the music therapy session			

### Interventions Within the Music

The open coding indicated that the interventions within the music could be categorized in two domains: active and receptive music therapy interventions. The axial coding led to several different types of active interventions, and two types of receptive interventions.

#### Active Interventions

The active interventions mentioned were categorized in a number of subcategories (see Table 1). For each of these subcategories, the coding led to several intervention characteristics, such as associated therapeutic goals, the musical instruments that were used, and the change factors that may possibly clarify the effect of the chosen intervention. Improvisation-based interventions concerned the first type of active interventions. Other types of active interventions included “singing and/or playing existing songs” and “songwriting and/or composing.” Lastly, the music therapists mentioned a variety of active interventions that referred to a certain music therapy method or approach. These were recognized as “other interventions” (see Table 1).

##### Improvisation

The music therapists agreed that improvisational method was one of the most often used active interventions. The second step of data-analysis showed that these improvisation-based interventions could be distinguished from each other by the level of structure, the applied improvisation techniques, the goal of the improvisation, and/or the used musical instruments. With respect to the process of selective coding, improvisation-based interventions for stress reduction could be subdivided in “free improvisation” and “structured improvisation.” Structured improvisation interventions refer to “freestyling,” “6/8-m improvisation,” “interplay on piano,” “expressing feelings in music,” “taking turns,” “mantra singing,” and “playing intrinsic tempo” (see Supplementary Appendix B for an extended description of each of these interventions). Most music therapists mentioned that they mainly preferred to use *free improvisation*, meaning that they start improvising with patients without using a pre-determined musical structure or certain rules for the music-making.

Participants appeared to identify two main therapeutic goals within free and structured improvisation: tension release and reaching direct relaxation. Synchronization is also mentioned as a goal, but the participants explained that they used it more as a sub goal. Specifically, free improvisation often starts with *synchronizing with the patient*, which can be achieved by attuning to the tempo and following the dynamics and intensity of the patients’ music-making, as can be seen in the quotation below. Subsequently, the music therapist tries to implement repetition and/or structure to allow the patient to slow down and ‘get a grip’ on their stress experience. Concerning synchronization as a sub goal, no specific musical instruments were mentioned.

“I often try to lower the stress level of the patient by first playing along with the patient, and then gradually lower the music tempo. As a therapist you hope that they synchronize with the music [participant 11, music therapist in Belgium].”

“For example, when the patient is playing the drums and is playing well enough, at least when you hear a rhythm, you can play along on the piano with only 4 chords [participant 3, music therapist in Netherlands].”

The first therapeutic goal for improvisation-based interventions concerned *releasing experienced tension*, which the music therapist tries to achieve by increasing the intensity of the music. One music therapist mentioned tension release as a goal for “freestyling,” which is classified as a more structured improvisation intervention (see Table 1) because of using a fixed beat and/or musical structure on which the freestyling (often through voice work) takes place. According to most of the participants, music improvisational methods focusing on tension release are characterized by starting with building up (accelerating) the music tempo and dynamics until a peak/climax is reached, and then slowly decreasing the tempo/dynamics. A potential change factor that was mentioned, which possibly contributes to achieving the goal, is that after an increase of (musical) tension, a decrease of this tension is experienced more “vividly” by the patient. Piano and percussion instruments were named as the musical instruments most often used in free improvisation interventions aiming at tension release.

“I first try to structure all the musicality that is there, a clear pulse, say, a clear rhythm. From there I often continue with the release of tension by playing faster and faster through differences in dynamics and in tempo [participant 9, music therapist in Germany].”

“I often use a form where you build up very slowly by making loud sounds and vocals, as a counterpart to a very structured intervention. Just a very free and fast way of expressing, in which the patient can connect to his/her feelings and then eventually release and discharge [participant 10, music therapist in Germany].”

Participants also noted *direct relaxation* as a therapeutic goal in improvisation-based interventions. Free improvisation aiming at relaxation is mainly characterized by the simplicity of the musical structures, such as playing in a low tonal register, using simple chord progressions, making minimal changes in music tempo or melodic intonation, and using easy-to-play musical instruments (e.g., monochord, wind chimes, ocean drum, rainmaker). In contrast to free improvisation aiming at the release of tension, free improvisation for direct relaxation is characterized by the use of a consistently low tempo, while changes in melody, harmonics, and tempo are kept to a minimum. The music therapists stated that it is precisely this simplicity of the musical improvisation that provides the patient with feelings of support and containment, both of which be regarded as certain *change factors* for achieving stress reduction in adults with MID. To reach relaxation by using free improvisation, three improvisation techniques (Bruscia, 1987) were regarded as helpful by some of the music therapists, i.e., “pacing,” “rhythmic grounding,” and “tonal centering.” The music therapists mentioned that they mainly use harmonic instruments (e.g., piano and guitar) and (melodic) percussion instruments (such as chimes and singing bowls).

“After a while, you lower the music tempo, and you will see that the patient is almost often following you in this and you see his stress level is lowering”[participant 3, music therapist in The Netherlands].”

“So, a solid beat is needed. That is how I see it. When patients with MID are highly tensioned, they have to experience themselves more, their own body, otherwise they lose it. By using a steady and solid beat, they feel it in their body, which is an important experience for them and helps them [participant 8, music therapist in Germany].”

##### Performing existing songs

Besides improvisation, “singing and/or playing of existing songs” was the type of active intervention most often mentioned by the music therapists. Interventions concerning singing by patients were singing well-known European-based cultural songs, singing the Greek Sirtaki (mentioned by one music therapist) and the use of the same opening song at the start of every session. The Greek Sirtaki intervention focuses on releasing tension and stimulating self-expression and is characterized by a (slow) increase of the music tempo, and clear rhythmic structures. The music therapist who often used this intervention clarified that the clearly defined rhythmic structure particularly promotes stress release in MID patients with externalizing behavioral patterns and can be seen as a possible change factor. With respect to the use of both “well-known songs” and “always using the same opening song,” the music therapists noted that it may provide a patient with feelings of safety due to potential linkages to cultural musical representations for patients. The presence of a repeating musical structure and familiarity can be regarded as possible change factors related to these effects (see Table 1).

##### Songwriting and composing

The active intervention category “song writing and/or composing” was mentioned only in the focus group held with the Dutch music therapists (*n* = 7). One of the participating music therapists clarified that music therapeutic songwriting can stimulate exposure to stress-causing situations and thereby help patients to release stress. Several other music therapists added that recordings of self-composed songs could also be helpful for patients to reduce stress by listening to the recordings outside of the music therapy session.

##### Other interventions

Some of the active interventions mentioned by the music therapists did not fit any of the previously described categories, but were based on a certain methodic framework (Hegi, Schuhmacher, or Ronnie-Gardiner) or the more protocol-oriented music therapy interventions (Neurologic Music Therapy [NMT]). One of the interventions mentioned was called “Eentoonssymphonie [One note symphony],” which originated from the clinical practice of the Dutch music therapist Berman (2016), and concerns music-making with just one note (see Supplementary Appendix B for more detailed descriptions of these active interventions).

### Receptive Interventions

In addition to the active interventions, the music therapists of this study also mentioned one receptive intervention, namely creating a personalized playlist for relaxation. Music therapists in all three focus groups referred to this receptive intervention for stress reduction. The personalized playlists can include both recorded musical improvisations and compositions made during the music therapy session(s), as well as pre-existing music of personal preference. The main goal focuses on “transfer,” meaning that patients can use this prerecorded music to lower their stress levels through relaxation outside of the music therapy sessions.

### Interventions Outside of the Music

The music therapists mentioned some stress-reducing interventions outside of the musical context, which are related to the therapeutic attitude of the music therapist. A majority of the Dutch focus group participants stated that creating a framework may offer support to the patient. This framework can be seen as a solid base of structure visible in both the interventions within the music and the way the music therapists interact with their patients. The music therapists indicated that after offering a structure or framework during a session, they simply “let the rest happen” and let the patient respond. One of the therapists stated that in some cases the music therapy session started with “doing nothing,” because often a starting point arises naturally, with which one can then continue working. The Belgian music therapists explained that they always base the starting point of their interventions on the patient’s needs.

Most of the music therapists considered the level of autonomy (self-control) they offered their patients, as an important aspect of the therapeutic attitude for reaching stress relief in patients with MID. The degree of autonomy provided to the patient depended on their individual needs and their capability to handle their self-control. One of the Dutch music therapists noted how important it is to take into account the patient’s strengths and limitations, such as not to have them feel overwhelmed or over-questioned. This sentiment was echoed by the German participants.

### Factors Influencing the Choice of the Intervention

Participants of two focus groups mentioned some factors that could possibly influence the stress-reducing effect of the applied music therapy interventions. To keep the results as close as possible to the initial data, we report a summary of the responses of the music therapists to this specific question.

The degree of intellectual impairment was considered as a factor for determining the choice of interventions. The Belgian music therapists stated that the degree of intellectual impairment (i.e., the IQ score) influences the effects of certain music therapy interventions, and therefore also influences their choice of intervention. They explained that when working with patients with MID from the upper segment of the IQ range, they often use rhythm-oriented interventions, such as drumming in the same beat or tapping along with the music on small percussion instruments. This is in contrast to interventions they offered to patients with MID in the lower segment of the IQ-range, which were characterized by a more multi-sensory approach and the use of tonal instruments.

Another factor possibly influencing the choice for a certain intervention was the mood or preference of the music therapist himself. For example, the music therapist may choose interventions that he/she is comfortable with and that makes him/her more relaxed and grounded as a therapist, which in turn is considered to have a positive influence on a patient.

Lastly, the patient’s preferences (like a specific music style, song, or instrument) were also mentioned as a factor that influenced the choice of intervention. Almost all the music therapists mentioned that they always tried to take the patient’s preferences into account. On the other hand, they tried to avoid the use of existing songs that were “too familiar,” as they noticed that patients tended to stick too much to the familiar lyrics instead of being aware of the musical experience.

## Discussion

In this study, we tried to explicate practice-based knowledge, also known as “tacit-knowledge,” about *how* music therapists can reduce stress in adults with MID during the music therapy session. The purpose of the present study was to describe music therapists’ experiences and perceptions on what works in their own practice, and provide an overview of music therapy interventions to generate an immediate stress-reducing effect in adults with MID. We discuss the implications of the results of this study by comparing them with the literature at large. Furthermore, we explore the facets of stress reduction, the musical components connected with such, and consider limitations of this study while providing implications for future practice, theory, and research.

### Two Ways to Reduce Stress in Music Therapy

Results of the present study distinguished two ways of intervening to reduce stress in adults with MID, which are related to the therapy goals mentioned by the music therapists. The achievement of (musical) *synchronization* with the patient, which can be regarded as a sub goal, often precedes working on one of the other two goals: *release of tension* or *direct relaxation*, which clearly leads to two different ways of intervening during music therapy.

The first way of intervening is related to the release of tension and aimed at attuning musically to the patient’s perceived tension/stress, which often results in music with an increased tempo and dynamics, and fast rhythms. After this, the music therapist further increases the intensity of the music, for example by accelerating the music tempo, so that the patient can release the felt tension. When the music intensity has come to a climax, the music therapist starts to slowly decrease the intensity of the music and actively guides the patient to decrease the music tempo to 60–80 bpm (beats per minute). This corresponds with previous research, which suggests that the tempo and dynamics of music are important for the experienced intensity of the music (Gabrielsson and Lindström, 2010). The second way of intervening is related to the achievement of direct relaxation. In contrast to the first way of intervening, the music therapist’s interventions are only focused on providing relaxation by synchronizing with the client’s music, and gradually decreasing the music tempo to 60–80 bpm and the volume to silence.

Both ways of intervening, aiming at the *release of tension* or *direct relaxation*, have common ground with the so-called “ISO-principle,” which refers to a music therapy technique by which the music is matched with the mood of a patient, then gradually altered to affect the desired mood state. This technique can also be used to affect physiological responses, such as heart rate and blood pressure (Davis et al., 2008).

### Musical Components

Within these two ways of stress reduction during music therapy, the *tempo* and the *dynamics* (also known as “loudness”) of the music can be considered as the most important musical components to reduce stress or tension in adult patients with MID. Studies suggest that music with a slow tempo can be considered to be one of the most significant determinants of audio-related effects on stress reduction (e.g., Iwanaga et al., 2005; Bernardi et al., 2006; Nilsson, 2008; Björkman et al., 2013; Jiang et al., 2016). Tempo and changes in tempo can influence different physiological and neurological responses, such as arousal, motor activity and motivation (Roth, 2014), this means that music with a slow tempo and steady rhythm may provide stress reduction by altering inherent body rhythms, such as heart rate (Thaut et al., 1999; Thaut and Hoemberg, 2014). Therefore, an increase in music tempo can lead to the increased activation of the nervous system, muscle tension and heart rate, whereas a decrease of the tempo can lead to muscle relaxation and a lowered heart rate resulting in more relaxation (Bernardi et al., 2006; Juslin and Västfjäll, 2008; Bringman et al., 2009; Nomura et al., 2013).

### Active Music Therapy Interventions

The present study makes a clear distinction between active and receptive music therapy interventions, which is in line with the music therapy literature (e.g., Bruscia, 2014; Wheeler, 2015; Magee, 2019). The results show that the participants of this study prefer the use of active music therapy interventions to reduce stress in adults with MID compared to receptive interventions. It is preferred to involve the patient in making music and/or singing to decrease the level of stress instead of offering receptive interventions, in which the patient only receives the music, such as listening to live or prerecorded music (Bruscia, 2014; Wheeler, 2015; Magee, 2019). A possible explanation could be that the interaction between patient and therapist during active interventions may lead to “affect attunement” because of the possibility to (co)-modulate, and (co)-regulate emotional states through the different musical components, such as rhythm, tempo or dynamics (Raglio et al., 2016; Aalbers et al., 2020). Similarly, findings of the present study suggest that synchronizing with patients’ music making and facilitating changes in musical expression by reducing tempo and volume, can lead to stress reduction. This is consistent with previous studies and literature, which describe music therapists’ use of synchronization techniques to attune to the patient for the purpose of change (e.g., Bruscia, 1987, 2014; Aalbers et al., 2019, 2020; Dvir et al., 2020).

Surprisingly, active improvisation interventions for stress reduction were used and few to none receptive interventions, whilst in reviews and meta-analyses on the effects of music or music therapy interventions on stress-related outcomes mainly receptive music listening interventions could be included (e.g., Pelletier, 2004; Bradt et al., 2013; De Witte et al., 2020a,b). The effects of music listening interventions are mainly related to the general influence of music on the stress response, whereas the effects of music therapy also can be explained by means of the therapeutic relationship, namely, through patient-therapist attunement by the use of music and the fact that music therapists individualize their interventions to meet the patients’ needs (Bradt and Dileo, 2014; Wheeler, 2015; De Witte et al., 2020a). Although none of these reviews and meta-analyses included adult patients with MID, it raises the question of whether music therapists should use receptive interventions more frequently because of their effects on stress reduction. On the other hand, there is evidence showing that action-based interventions are very suitable to adults with MID (Cuijpers et al., 2007; Didden et al., 2016), which could favor active music therapy interventions.

### Music Therapeutic Improvisation for Stress Reduction

Results show that the participants of this study commonly used active improvisation interventions for stress reduction, while their use of receptive intervention processes was either limited or non-existent. Literature suggests that improvisational methods are highly common in music therapy; within musical improvisation, patient(s) and therapist improvise on musical instruments they have chosen and play together freely or with a given structure (Wigram, 2004; Gold et al., 2009; MacDonald and Wilson, 2014). Gold et al. (2009) emphasize in their review that improvisational music therapy techniques can be regarded as the most important interventions for achieving social interaction in active music therapy. In addition, musical improvisation can also be seen as a mode of self-expression, where the expressive character of musical interactions enables the release of difficult or repressed emotions (Burns et al., 2001; Gilboa et al., 2006; MacDonald and Wilson, 2014), which corresponds with the therapy goal of achieving tension release, found in this study. However, researchers reported that the use of improvisation may also evoke stressful experiences, necessitating the presence of an educated and qualified music therapist to attune from moment to moment to the patient’s needs (Magee, 2019; De Witte et al., 2020a; Aalbers et al., under revision). Thereby, the understanding of the links between the music-related mechanisms and synchronization may help music therapists to make informed decisions on how to use musical improvisation to lower the patient’s stress levels (Moore, 2013; MacDonald and Wilson, 2014; De Witte et al., 2020a; Aalbers et al., under revision).

### Change Factors

Results of this study identified some factors that may contribute to the explanation of the perceived effects of specific music therapy interventions for stress reduction in adults with MID. In the body of literature, these factors are often described as “change factors (Lambert, 2013; Cuijpers et al., 2019).” An important change factor found for promoting stress release during music therapy in adults with MID concerns the amount of simplicity involved in the chosen interventions. Examples of this are the use of mantra techniques, which have a repetitive character, or the choice for a simple harmonic structure, which seems to provide support. This fits the concept of the technique Vocal Holding, in which the music therapist is vocally following, supporting, and mirroring the patient while accompanying with only two piano chords, to create an atmosphere that is safe and predictable during improvising (Monti and Austin, 2018). Besides, music therapy literature indicates that “lullabies” with characteristics very similar to the mantra-based interventions, also generate stress-reducing effects, which is attributed to the simplicity of the musical components, such as a slow and steady tempo, a repetitive and simple rhythm, and just a few different notes (Standley, 2003; Friedman et al., 2010).

The importance of implementing a solid structure is also emphasized when reviewing the interventions outside of the music, which are focused on establishing a clear, predictable and above all a safe framework for the patient, giving the patient the support and confidence to play, experiment and express themselves within the offered framework.

Strong similarities can be seen between the possible change factors found in this study and the general characteristics that can positively influence the quality of care or treatment of people with MID. Literature shows that psychological treatment for people with MID may benefit from a clear structure and predictability (Ten Wolde et al., 2006; Došen, 2007; Elias et al., 2009). Creating favorable environmental conditions, such as a predictable course of events in daily life or a clear structure of the therapeutic session based on small feasible steps, can enhance feelings of safeness and reduce stress in people with MID, which will benefit treatment (Didden, 2007). These important factors, which can be seen as treatment guidelines for adults with MID, are in line with the possible change factors found in this study, such as the creation of a predictable musical structure.

### Strengths and Limitations of the Present Study

In order to show international practice-based knowledge, the current study was conducted in three different countries, since results related to the specific context of one country may not be relevant to other countries. Firstly, international definitions of MID and subsequent clinical care for adults with MID differs between countries (Moonen, 2017). Secondly, music therapy education differs among countries with regard to music therapy methods and general theories. As only three countries were involved, each with close proximity to each other, results are unlikely to be generalizable. Replication of this study across different continents is strongly recommended.

With respect to some of the other results, more research is needed to be able to generalize findings, especially regarding change factors. The initial research questions focused on how and in what way music therapists could lower stress levels of patients with MID instead of exploring *why* interventions can lead to stress reduction. Data-analysis revealed some of these possible change factors, and we decided to report them (see Table 1). In future research, we suggest including research questions specifically addressing “change factors” or “mechanisms of change,” because this may yield important knowledge on both the development and the evaluation of music therapy interventions for stress reduction in adults with MID.

Several techniques were applied to enhance the quality of the present study. The use of an interview guide, a codebook, the iterative process and the ‘constant comparison’ method during data analysis can be seen as a strength of this study, because it helps to ensure that the results stay as close to the original data as possible. We found this particularly important because this is the first study in which music therapists in clinical practice were interviewed about their music therapy interventions to reduce stress in patients with MID.

### Implications for Clinical Practice

The findings of this study contribute to both the body of practice-based knowledge on which specific interventions can be used by music therapists to reduce stress in patients with MID, and to the knowledge base of experiential interventions for people with MID in addition to the cognitive-oriented approaches, such as “cognitive behavioral therapy” [CBT].

Firstly, results show two main ways to reach stress reduction. Although both ways are intended to lead to the same result (stress reduction), the order of intervention steps and/or the way in which the music therapist intervenes on the basis of musical components, may be different. Therefore, it is important that music therapists, prior to the intervention, have a clear understanding of the needs of their patient and the most fitting goal (stress release or direct relaxation), so that the most suitable interventions can be applied.

Secondly, the present study shows that improvisation is the most commonly used intervention by music therapists to reduce stress in adults with MID (see Table 1 for an overview of the different types of the mentioned improvisation-based interventions). When using improvisation-based interventions to reduce stress in patients with MID, according to the present study, there are some key elements that can positively influence the effect of the intervention. Namely, the use of simple musical structures, which means that the improvisation has a repetitive and predictable character. Also, synchronizing with the patient during improvising, whether as a stand-alone goal or as a step-in working on the goals “stress release” or “direct relaxation,” helps the music therapist to attune to the patient’s needs from moment-to-moment. This may enhance feelings of safety, which is especially important for the clinical treatment of adults with MID (Didden, 2007) and can therefore increase the effect of improvisation.

Thirdly we believe that the results of the present study may provide a useful basis for the further development of more explicit music therapy intervention descriptions for stress reduction in patients with MID. According to the previous work of Robb et al. (2011), intervention descriptions of music therapy should be tailored to the target population, and realistic therapy goals as well as desired outcomes should be defined. Moreover, Hanson-Abromeit (2015) adds the importance of defining the purpose and intention of each musical element in the descriptions of music therapy interventions, such as the specific use of musical techniques within musical improvisation.

Fourthly, with the findings of the current study, we kindly recommend that other health care professionals responsible for referral to therapy (e.g., psychologists, physicians, health care coordinators) will consider experience-based interventions, such as music therapy, since both literature and results of the present study show that they are well suited to the needs and capabilities of adults with MID (Didden et al., 2016; De Witte et al., 2017). Indeed, from neurological perspectives, music is intrinsically motivating, drives motor function and elicits emotional responses (Thaut and Hoemberg, 2014; Koelsch et al., 2016). Moreover, according to the music therapists of the present study, it is the structure of music which provides a sense of safety, which benefits treatment in adults with MID. However, although psychologists often recognize that the use of music in daily life helps to manage stress, they still appreciate additional knowledge about why and when a music therapist should be involved in the treatment of their patients (Magee, 2019; Aalbers et al., under revision). Our findings not only provide more insight into the different types of music therapy interventions for stress reduction, but also into the related goals, techniques, and change factors. This helps to understand when, why, and how music therapy can be effective for stress reduction, which reinforces referral to music therapy on the basis of substantive grounds. In addition, the results of the present study demonstrate the need for a clear and thorough assessment of patient with MID before any therapeutic intervention should be initiated.

### Recommendations for Future Research

The findings of this study may help to implement music therapy interventions for stress reduction in adults with MID, but more research is needed to assess the effectiveness and applicability of these interventions. It would be relevant to replicate this and future studies in other parts of the world. In future research it is recommended to add research questions on therapeutic change factors, which may help to explain *why* the interventions used possibly lead to stress reduction. We also strongly recommend a study employing a systematic review methodology – such as a meta-synthesis – to analyze music therapy literature regarding possible change factors, as the number of empirical studies has increased in recent years. Finally, we would welcome the development of standardized intervention descriptions, like music therapy protocols, to enhance treatment fidelity, enabling more robust research on the effects of music therapy. Detailed intervention descriptions within research are essential for replication and translation of music therapy interventions to clinical practice (Stouffer et al., 2007; Robb et al., 2011; Hoffmann et al., 2014).

## Data Availability Statement

The raw data supporting the conclusions of this article will be made available by the authors, without undue reservation.

## Ethics Statement

The study was approved by the Ethical Research Committee of the HAN University of Applied Sciences in Netherlands (ref. no: 198.09/20). Written informed consent to participate in this study was provided by the participants.

## Author Contributions

All authors listed have made a substantial, direct and intellectual contribution to the work, and approved it for publication.

## Conflict of Interest

The authors declare that the research was conducted in the absence of any commercial or financial relationships that could be construed as a potential conflict of interest.

## Footnotes

1 By common Dutch definition adults with an IQ 50–70 as well as with an IQ 70–85 in combination with a disability in adaptive skills are considered to have a MID. In this article the abbreviation MID is therefore used to include people with what is often referred to as a “borderline intellectual disability” (Kaal et al., 2015).

2 In this study, the term “intervention” refers to a specified therapeutic action or a process of intervening characterized by a structured and coherent collection of therapeutic actions.

## References

[B1] Aalbers S. Spreen M. Pattiselanno K. Verboon P. Vink A. van Hooren S. (2020). Efficacy of emotion-regulating improvisational music therapy to reduce depressive symptoms in young adult students: a multiple-case study design. *Arts Psychother.* 71:101720 10.1016/j.aip.2020.101720

[B2] Aalbers S. Vink A. Freeman R. E. Pattiselanno K. Spreen M. van Hooren S. (2019). Development of an improvisational music therapy intervention for young adults with depressive symptoms: an intervention mapping study. *Arts Psychother.* 65:101584 10.1016/j.aip.2019.101584

[B3] Aalbers S. Vink A. de Witte M. Patisselanno K. Spreen M. van Hooren S. (under revision). *Emotion-regulating improvisation music therapy for adult students with depressive symptoms: A process-evaluation using a multiple-case study design.*

[B4] Abbaszadeh M. Sardoie G. (2016). Compare academic self-efficacy and self-regulation among students with learning disorder and without learning disorder in normal elementary schools (fourth and fifth grade) of Kerman. *Biomed. Pharmacol. J.* 9 751–759. 10.13005/bpj/999

[B5] Agres K. Schaefer R. Volk A. Van Hooren S. Holzapfel A. Dalla B. (under revision). *Music, Computing, and Health: A roadmap for the current and future roles of music technology for healthcare and well-being.* 10.13005/bpj/999

[B6] Aigen K. (1999). Revisiting edward: an exemplar of tacit knowledge. *Nord. J. Music Ther.* 8 89–95. 10.1080/08098139909477957

[B7] Aldwin C. M. (2007). *Stress, Coping, and Development: An Integrative Perspective*, 2nd Edn New York, NY: Guilford Press.

[B8] American Music Therapy Association [AMTA] (2018). *Definition and Quotes About Music Therapy. What is Music Therapy?* Silver Spring, MD: AMTA.

[B9] American Psychiatric Association [APA] (2013). “Intellectual disability (intellectual developmental disorder),” in *Proceedings of the Diagnostic and Statistical Manual of Mental Disorders*, 5th Edn, (Arlington, VA: American Psychiatric Association), 33–41.

[B10] American Psychological Association [APA] (2017). *Stress in America: Coping with change.* Washington, DC: APA.

[B11] Australian Psychological Society [APS] (2015). *Stress & Wellbeing: How Australians are Coping With Life: The Findings of the Australian Psychological Society Stress and Wellbeing in Australia Survey 2015.* Melbourne: APS.

[B12] Bernardi L. Porta C. Sleight P. (2006). Cardiovascular, cerebrovascular, and respiratory changed induced by different types of music in musicians and non-musicians: the importance of silence. *BMJ Heart* 92 445–452. 10.1136/hrt.2005.064600 16199412 PMC1860846

[B13] Björkman I. Karlsson F. Lundberg A. Frisman G. H. (2013). Gender differences when using sedative music during colonoscopy. *Gastroenterol. Nurs.* 36 14–20. 10.1097/SGA.0b013e31827c4c80 23364361

[B14] Bradt J. Dileo C. (2014). Music interventions for mechanically ventilated patients. *Cochrane Database Syst. Rev.* 2014:CD006902. 10.1002/14651858.CD006902.pub3 25490233 PMC6517146

[B15] Bradt J. Dileo C. Potvin N. (2013). Music for stress and anxiety reduction in coronary heart disease patients. *Cochrane Database Syst. Rev.* 2013:CD006577. 10.1002/14651858.CD006577.pub3 24374731 PMC8454043

[B16] Bradt J. Potvin N. Kesslick A. Shim M. Radl D. Schriver E. (2015). The impact of music therapy versus music medicine on psychological outcomes and pain in cancer patients: a mixed methods study. *Support. Care Cancer* 23 1261–1271. 10.1007/s00520-014-2478-7 25322972

[B17] Brennand R. Schepman A. Rodway P. (2011). Vocal emotion perception in pseudo-sentences by secondary-school children with autism spectrum disorder. *Res. Autism Spect. Disord.* 5 1567–1573. 10.1016/j.rasd.2011.03.002

[B18] Bringman H. Giesecke K. Thörne A. Bringman S. (2009). Relaxing music as pre-medication before surgery: a randomized controlled trial. *Acta Anaesthesiol. Scand.* 53 759–764. 10.1111/j.1399-6576.2009.01969.x 19388893

[B19] Bruscia K. E. (1987). *Improvisational Models of Music Therapy.* Springfield, IL: Charles C Thomas.

[B20] Bruscia K. E. (2014). *Defining music therapy.* 3rd edn University Park, IL: Barcelona Publishers.

[B21] Burns S. Harbuz M. Hucklebridge F. Bunt L. (2001). A pilot study into the therapeutic effects of music therapy at a cancer help center. *Alternat. Ther. Health Med.* 7 48–56. 11191042

[B22] Casey G. (2017). Stress and disease. *Kai Tiaki Nursing New Zeal.* 23 20–24. 10.1002/3527609156.ch2

[B23] Chanda M. L. Levitin D. J. (2013). The neurochemistry of music. *Trends Cogn. Sci.* 17 179–193. 10.1016/j.tics.2013.02.007 23541122

[B24] Charmaz K. (2003). “Grounded theory: Objectivist and constructivist methods,” in *Strategies for qualitative inquiry*, 2nd Edn, eds Denzin N. K. Lincoln Y. S. (Thousand Oaks, CA: Sage Publications).

[B25] Cho J. Y. Lee E. H. (2014). Reducing confusion about grounded theory and qualitative content analysis: similarities and differences. *Qual. Rep.* 19:1.

[B26] Corbin J. Strauss A. (2008). *Basics of Qualitative Research.* Los Angeles, CA: Sage Publications.

[B27] Creswell J. W. (1998). *Qualitative Inquiry and Research Design*: *Choosing Among Five Traditions*, 1st Edn Los Angeles, CA: Sage Publications.

[B28] Cuijpers P. Reijnders M. Huibers M. J. H. (2019). The role of common factors in psychotherapy outcomes. *Annu. Rev. Clin. Psychol.* 15 207–231. 10.1146/annurev-clinpsy-050718-095424 30550721

[B29] Cuijpers P. Van Straten A. Warmerdam L. (2007). Behavioral activation treatments of depression: a meta-analysis. *Clin. Psychol. Rev.* 27 318–326. 10.1016/j.cpr.2006.11.001 17184887

[B30] Davis W. B. Gfeller K. E. Thaut M. H. (2008). *An Introduction to Music Therapy: Theory and Practice*, 3rd Edn Silver Spring, MD: American Music Therapy Association.

[B31] De Witte M. Pinho A. Stams G. J. Moonen X. Bos A. van Hooren S. (2020a). Music therapy for stress reduction: a systematic review and meta-analysis. *Health Psychol. Rev.* 10.1080/17437199.2020.1846580 33176590

[B32] De Witte M. Spruit A. van Hooren S. Moonen X. Stams G. J. (2020b). Effects of music interventions on stress-related outcomes: a systematic review and two meta-analyses. *Health Psychol. Rev.* 14 294–324. 10.1080/17437199.2019.1627897 31167611

[B33] De Witte M. D. Bellemans T. Tukker K. van Hooren S. A. H. (2017). “Vaktherapie,” in *Handboek emotionele ontwikkeling en verstandelijke beperking* [Handbook emotional development and intellectual dissability], eds de Bruijn J. Vonk J. van den Broek A. (Amsterdam: Boom), 277–290.

[B34] Denzin N. K. Lincoln Y. S. (2005). “Paradigms and perspectives in contention,” in *The SAGE Handbook of Qualitative Methods in Health Research*, eds Bourgeault I. Dingwall R. de Vries R. (London: Sage Publications), 183–190.

[B35] Didden R. (2007). “Effectieve behandeling van jeugdigen en volwassenen met een lichte verstandelijke beperking: een beschouwing [Effective treatment of adolescents and adults with a mild intellectual dissability: a review],” in *Met het oog op behandeling: effectieve behandeling van gedragsstoornissen van mensen met een licht verstandelijke beperking*, eds Didden R. Moonen X. (Utrecht: Landelijk Kenniscentrum LVG), 129–135.

[B36] Didden R. Embregts P. van der Toorn M. Laarhoven N. (2009). Substance abuse, coping strategies, adaptive skills and behavioral and emotional problems in patients with mild to borderline intellectual disability admitted to a treatment facility: a pilot study. *Res. Dev. Disabil.* 30 927–932. 10.1016/j.ridd.2009.01.002 19217753

[B37] Didden R. Lindsay W. R. Lang R. Sigafoos J. Dab S. Wierma J. (2016). “Aggressive behavior,” in *Clinical Handbook of Evidence-Based Practices for Individuals With Intellectual and Developmental Disabilities*, ed. Singh N. N. (New York, NY: Springer), 727–750.

[B38] Dörnyei Z. (2007). *Research Methods in Applied Linguistics.* New York, NY: Oxford University Press.

[B39] Došen A. (2007). Integrative treatment in persons with intellectual disability and mental health problems. *J. Intell. Disabil. Res.* 51 66–74. 10.1111/j.1365-2788.2006.00868.x 17181604

[B40] Dulin P. L. Hanson B. L. King D. K. (2013). Perceived control as a longitudinal moderator of late-life stressors on depressive symptoms. *Aging Mental Health* 17 718–723. 10.1080/13607863.2013.784956 23550624

[B41] Dvir T. Lotan N. Viderman R. Elefant C. (2020). The body communicates: Movement synchrony during music therapy with children diagnosed with ASD. *Arts Psychother.* 69:101658 10.1016/j.aip.2020.101658

[B42] Elias C. Seebregts A. Swennenhuis P. Bouma G. (2009). *Traumabehandeling bij kinderen en jongeren met een licht verstandelijke beperking. Naar een methodiek voor de behandeling van getraumatiseerde kinderen en jongeren met een licht verstandelijke beperking* [The treatment of trauma in children and adolescents with a mild intellectuel disability. After a method for the treatment of traumatised children and adolescents with a mild intellectual disability] Tilburg: Fontys Hoogeschool.

[B43] Emerson E. (2003). Mothers of children and adolescents with intellectual disability: social and economic situation, mental health status, and the self-assessed social and psychological impact of the child’s difficulties. *J. Intell. Disabil. Res.* 47 385–399. 10.1046/j.1365-2788.2003.00498.x 12787168

[B44] Etikan I. Musa S. A. Alkassim R. S. (2016). Comparison of convenience sampling and purposive sampling. *Am. J. Theor. Appl. Statist.* 5 1–4. 10.11648/j.ajtas.20160501.11

[B45] Everly G. S. Lating J. M. (2019). “Resilience: The final frontier,” in *A clinical guide to the treatment of the human stress response*, eds Everly G. S. Lating J. M. (New York, NY: Springer), 175–187. 10.1007/978-1-4939-9098-6_8

[B46] Friedman S. H. Kaplan R. S. Rosenthal M. B. Console P. (2010). Music therapy in perinatal psychiatry: use of lullabies for pregnant and postpartum women with mental illness. *Music Med.* 2 219–225. 10.1177/1943862110379584

[B47] Gabrielsson A. Lindström E. (2010). “The role of structure in the musical expression of emotions,” in *Handbook of Music and Emotion: Theory, Research, Applications*, eds Juslin P. N. Sloboda J. A. (Oxford: Oxford University Press), 367–400. 10.1093/acprof:oso/9780199230143.003.0014

[B48] Gilboa A. Bodner E. Amir D. (2006). Emotional communicability in improvised music: the case of music therapists. *J. Music Ther.* 43 198–225. 10.1093/jmt/43.3.198 17037951

[B49] Gold C. Solli H. P. Krüger V. Lie S. A. (2009). Dose–response relationship in music therapy for people with serious mental disorders: systematic review and meta-analysis. *Clin. Psychol. Rev.* 29 193–207. 10.1016/j.cpr.2009.01.001 19269725

[B50] Grocke D. Wigram T. (2006). *Receptive Methods in Music Therapy: Techniques and Clinical Applications for Music Therapy Clinicians, Educators and Students.* London: Jessica Kingsley Publishers.

[B51] Hamelin J. Travis R. Sturmey P. (2013). Anger management and intellectual disabilities: a systematic review. *J. Mental Health Res. Intell. Disabil.* 6 60–70. 10.1080/19315864.2011.637661

[B52] Hanson-Abromeit D. (2015). A conceptual methodology to define the therapeutic function of music. *Music Ther. Perspect.* 33 25–38. 10.1093/mtp/miu061

[B53] Hartley S. L. MacLean W. E. Jr. (2009b). Stressful social interactions experienced by adults with mild intellectual disability. *Am. J. Intell. Dev. Disabil.* 114 71–84. 10.1352/2009.114.71-84 19391674 PMC2831777

[B54] Hartley S. L. Maclean W. E. Jr. (2009a). Depression in adults with mild intellectual disability: role of stress, attributions, and coping. *Am. J. Intell. Dev. Disabil.* 114 147–160. 10.1352/1944-7588-114.3.147 19374469 PMC2831402

[B55] Hatton C. Emerson E. (2004). The relationship between life events and psychopathology amongst children with intellectual disabilities. *J. Appl. Res. Intell.l Disabil.* 17 109–117. 10.1111/j.1360-2322.2004.00188.x

[B56] Heyman M. Hauser-Cram P. (2015). Negative life events predict performance on an executive function task in young adults with developmental disabilities. *J. Intell. Disabil. Res.* 59 746–754. 10.1111/jir.12181 25559271

[B57] Hoffmann T. C. Glasziou P. P. Boutron I. Milne R. Perera R. Moher D. (2014). Better reporting of interventions: template for intervention description and replication (TIDieR) checklist and guide. *Bmj* 348:g1687. 10.1136/bmj.g1687 24609605

[B58] Holahan C. J. Moos R. H. Holahan C. K. Brennan P. L. Schutte K. K. (2005). Stress generation, avoidance coping, and depressive symptoms: a 10-year model. *J. Consult. Clin. Psychol.* 73 658–666. 10.1037/0022-006X.73.4.658 16173853 PMC3035563

[B59] Hooper J. Wigram T. Carson D. Lindsay B. (2008a). A review of the music and intellectual disability literature (1943–2006) part one—descriptive and philosophical writing. *Music Ther. Perspect.* 26 66–79. 10.1093/mtp/26.2.66

[B60] Hooper J. Wigram T. Carson D. Lindsay B. (2008b). A review of the music and intellectual disability literature (1943–2006) part two—experimental writing. *Music Ther. Perspect.* 26 80–96. 10.1093/mtp/26.2.80

[B61] Hooper J. Wigram T. Carson D. Lindsay B. (2011). The practical implication of comparing how adults with and without intellectual disability respond to music. *Br. J. Learn. Disabil.* 39:22 10.1111/j.1468-3156.2010.00611.x

[B62] Hoyle J. N. McKinney C. H. (2015). Music therapy in the bereavement of adults with intellectual disabilities: a clinical report. *Music Ther. Perspect.* 33 39–44. 10.1093/mtp/miu051

[B63] Hulbert-Williams L. Hastings R. P. (2008). Life events as a risk factor for psychological problems in individuals with intellectual disabilities: a critical review. *J. Intell. Disabil. Res.* 52 883–895. 10.1111/j.1365-2788.2008.01110.x 18671807

[B64] Iwanaga M. Kobayashi A. Kawasaki C. (2005). Heart rate variability with repetitive exposure to music. *Biol. Psychol.* 70 61–66. 10.1016/j.biopsycho.2004.11.015 16038775

[B65] Jiang J. Rickson D. Jiang C. (2016). The mechanism of music for reducing psychological stress: music preference as a mediator. *Arts Psychotherapy* 48 62–68. 10.1016/j.aip.2016.02.002

[B66] Juslin P. N. Västfjäll D. (2008). Emotional responses to music: the need to consider underlying mechanisms. *Behav. Brain Sci.* 31 559–575. 10.1017/S0140525X08005293 18826699

[B67] Kaal H. L. Moonen X. M. H. Nijman H. L. I. (2015). Identifying offenders with an intellectual disability in detention in The Netherlands. *J. Intell. Disabil. Off. Behav.* 6 94–101. 10.1108/JIDOB-04-2015-0008

[B68] Kitzinger J. (2013). “Using focus group to understand experiences of health and illness,” in *Understanding and Using Health Experiences. Improving Patient Care*, eds Ziebland S. Coulter A. Calabrese J. D. Locock L. (Oxford: Oxford University Press), 49–59.

[B69] Koelsch S. Boehlig A. Hohenadel M. Nitsche I. Bauer K. Sack U. (2016). The impact of acute stress on hormones and cytokines, and how their recovery is affected by music-evoked mood. *Sci. Rep.* 6:23008. 10.1038/srep23008 27020850 PMC4810374

[B70] Lambert M. J. (2013). *The Efficacy and Effectiveness of Psychotherapy. In Bergin and Garfield’s Handbook of Psychotherapy and Behavior Change*, 6th Edn Hoboken, NJ: John Wiley, 169–218.

[B71] Liamputtong P. (2011). *Focus group Methodology*. *Principles and Practice.* London: Sage Publishers.

[B72] Litosseliti L. (2003). *Using Focus Groups in Research.* London: Continuum.

[B73] Lunsky Y. (2008). The impact of stress and social support on the mental health of individuals with intellectual disabilities. *Salud Pública de México* 50 151–153. 10.1590/s0036-36342008000800007 18470342

[B74] Lunsky Y. Benson B. A. (2001). Association between perceived social support and strain, and positive and negative outcome for adults with mild intellectual disability. *J. Intell. Disabil. Res.* 45 106–114. 10.1046/j.1365-2788.2001.00334.x 11298249

[B75] MacDonald R. A. Wilson G. B. (2014). Musical improvisation and health: a review. *Psychol. Well Being* 4 1–18. 10.1186/s13612-014-0020-9

[B76] Magee W. L. (2019). Why include music therapy in a neuro-rehabilitation team. *Adv. Clin. Neurosci. Rehabil.* 19 10–12.

[B77] McAdam R. Mason B. McCrory J. (2007). Exploring the dichotomies within the tacit knowledge literature: towards a process of tacit knowing in organizations. *J. Knowl. Manag.* 11 43–59. 10.1108/13673270710738906

[B78] Meila A. M. (2017). *Exploring the Use of Music Interventions on Emotion Competence in Individuals With Special Needs: A Systematic Review.* Open Access Theses Master of Music (MM), University of Miami, Coral Gables, FL.

[B79] Monti E. Austin D. (2018). The dialogical self in vocal psychotherapy. *Nordic J. Music Ther.* 27 158–169.

[B80] Moonen X. Didden R. (2014). “Effectieve methoden in de ondersteuning aan mensen met een verstandelijke beperking [Effective methods in the support of people with an intellectual disability],” in *Handboek verstandelijke beperking 24 succesvolle methoden*, eds Twint B. de Bruijn J. (Amsterdam: Uitgeverij Boom).

[B81] Moonen X. M. H. (2017). (H)erkennen en waarderen: over het (h) erkennen van de noden mensen met licht verstandelijke beperkingen en het bieden van passende ondersteuning [Recognize, acknowlegde and appreciate: about recognizing/acknowledging the needs of people with a mild intellectual disability and offering them appropriate support]. *Nederlands tijdschrift voor de zorg aan mensen Met Verstandelijke Beperkingen* 43 163–176.

[B82] Moore K. S. (2013). A systematic review of the neural effects of music on emotion regulation: implications for music therapy practice. *J. Music Ther.* 50 198–242. 10.1093/jmt/50.3.198 24568004

[B83] Moretti F. van Vliet L. Bensing J. Deledda G. Mazzi M. Rimondini M. (2011). A standardized approach to qualitative content analysis of focus group discussions from different countries. *Patient Educ. Couns.* 82 420–428. 10.1016/j.pec.2011.01.005 21292424

[B84] Morgan D. Scannell A. (1998). *Planning Focus Groups.* Thousands Oaks, CA: Sage Publishers.

[B85] Morgan D. L. (1997). *The Focus Group Guidebook*, Vol. 1 Thousand Oaks, CA: Sage Publications.

[B86] Nandy B. R. Sarvela P. D. (1997). Content analysis reexamined: a relevant research method for health education. *Am. J. Health Behav.* 21 222–234.

[B87] Nilsson U. (2008). The anxiety- and pain-reducing effects of music interventions: a systematic review. *Assoc. Periopera Regist. Nurses J.* 87 780–807. 10.1016/j.aorn.2007.09.013 18395022

[B88] Nomura S. Yoshimura K. Kurosawa Y. (2013). A pilot study on the effect of music-heart beat system on human heart activity. *J. Med. Inform. Technol.* 22 251–256.

[B89] Patterson C. W. Williams J. Jones R. (2019). Third-wave therapies and adults with intellectual disabilities: a systematic review. *J. Appl. Res. Intell. Disabil.* 32 1295–1309. 10.1111/jar.12619 31094063

[B90] Patton M. Q. (2014). *Qualitative Research & Evaluation Methods: Integrating Theory and Practice.* Thousand Oaks, CA: Sage Publications.

[B91] Pelletier C. L. (2004). The effect of music on decreasing arousal due to stress: a meta-analysis. *J. Music Ther.* 41 192–214. 10.1093/jmt/41.3.192 15327345

[B92] Petri G. Beadle-Brown J. Bradshaw J. (2020). Redefining self-advocacy: a practice theory-based approach. *J. Poli. Pract. Intell. Disabil.* 17 207–218. 10.1111/jppi.12343

[B93] Polanyi M. (2009). *The Tacit Dimension.* Chicago, IL: University of Chicago press.

[B94] Raglio A. Galandra C. Sibilla L. Esposito F. Gaeta F. Di Salle F. (2016). Effects of active music therapy on the normal brain: fMRI based evidence. *Brain Imaging Behav.* 10 182–186. 10.1007/s11682-015-9380-x 25847861

[B95] Riley K. E. Park C. L. (2015). How does yoga reduce stress? A systematic review of mechanisms of change and guide to future inquiry. *Health Psychol. Rev.* 9 379–396. 10.1080/17437199.2014.981778 25559560

[B96] Robb S. L. Carpenter J. S. Burns D. S. (2011). Reporting guidelines for music-based interventions. *J. Health Psychol.* 16 342–352. 20709884 10.1177/1359105310374781 PMC3141224

[B97] Roth E. (2014). “Clinical improvisation in neurologic music therapy,” in *Handbook of Neurologic Music Therapy*, eds Thaut M. H. Hoemberg V. (Oxford: Oxford University Press), 24–46.

[B98] Schuengel C. Janssen C. G. (2006). People with mental retardation and psychopathology: stress, affect regulation and attachment: a review. *Int. Rev. Res. Mental Retard.* 32 229–260. 10.1016/S0074-7750(06)32008-3

[B99] Schumacher K. (2001). “The physical-emotional development within music therapy of children suffering from severe contact deficiencies,” in *Summary of the lectures Music – Emotion – Therapy 2001. Music & Medicine*. Vienna: Herbert von Karajan Centre.

[B100] Schumacher K. Calvet C. (2008). *Synchronization. Music Therapy With Children on the Autistic Spectrum.* Göttingen: Vandenhoeck & Ruprecht.

[B101] Scott H. M. Havercamp S. M. (2014). Mental health for people with intellectual disability: the impact of stress and social support. *Am. J. Intell. Dev. Disabil.* 119 552–564. 10.1352/1944-7558-119.6.552 25354124

[B102] Seyed S. Salmani M. Motahari Nezhad F. Noruzi R. (2017). Self-efficacy, achievement motivation, and academic progress of students with learning disabilities: a comparison with typical students. *Middle East J. Rehabil Health* 4:e44558 10.5812/mejrh.44558

[B103] Smeijsters H. Vink A. (2006). Research in practice. *Music Ther. Today* 7 781–838.

[B104] Standley J. (2003). *Music Therapy with Premature Infants.* Silver Spring, MD: American Music Therapy Association.

[B105] Stige B. (2015). The practice turn in music therapy theory. *Music Ther. Perspect.* 33 3–11. 10.1093/mtp/miu050

[B106] Stouffer J. W. Shirk B. J. Polomano R. C. (2007). Practice guidelines for music interventions with hospitalized pediatric patients. *J. Pediatr. Nurs.* 22 448–456. 10.1016/j.pedn.2007.04.011 18036465

[B107] Ten Wolde A. C. Le Grand B. Slagter J. Storm M. (2006). *Vaardig en veilig: Behandeling van sterk gedragsgestoorde licht verstandelijk gehandicapte mensen met risicovol gedrag. Kenmerken van de doelgroep, consequenties van behandeling en de uitwerking hiervan in gespecialiseerde behandelprogramma’s* [Skillful and safe: The treatment of patients with mild intellectual disabilities with risky behavior. Characteristics of the target population, consequences of treatment and the effects on specialized treatment programs] Boschoord: Hoeve Boschoord.

[B108] Thaut M. H. Hoemberg V. (eds) (2014). *Handbook of Neurologic Music Therapy.* Oxford: Oxford University Press.

[B109] Thaut M. H. Kenyon G. P. Schauer M. L. McIntosh G. C. (1999). The connection between rhythmicity and brain function. *IEEE Eng. Med. Biol. Mag.* 18 101–108. 10.1109/51.752991 10101675

[B110] Valerio M. A. Rodriguez N. Winkler P. Lopez J. Dennison M. Liang Y. (2016). Comparing two sampling methods to engage hard-to-reach communities in research priority setting. *BMC Med. Res. Methodol.* 16:146. 10.1186/s12874-016-0242-z 27793191 PMC5084459

[B111] van Bruggen-Rufi M. Vink A. Achterberg W. Roos R. (2018). Improving quality of life in patients with Huntington’s disease through music therapy: a qualitative explorative study using focus group discussions. *Nordic J. Music Ther.* 27 44–66. 10.1080/08098131.2017.1284888

[B112] Vaughn S. Schumm J. S. Sinagub J. M. (1996). *Focus Group Interviews in Education and Psychology.* Thousand Oaks, CA: Sage Publishers 10.4135/9781452243641

[B113] Watson D. M. (2002). Drumming and improvisation with adult male sexual offenders. *Music Therapy Perspect*. 20 105–111. 10.1093/mtp/20.2.105

[B114] Wheeler B. L. (ed.) (2015). *Music Therapy Handbook.* New York, NY: Guilford Publications.

[B115] Wigram T. (2004). *Improvisation: Methods and Techniques for Music Therapy Clinicians, Educators and Students.* London: Jessica Kingsley Publishers.

[B116] World Health Organization [WHO] (2010). *Global Health Diplomacy: Negotiating Health in the 21st Century.* Geneva: WHO.

