# Supplementary material for: Music Therapy Interventions for Stress Reduction in Adults With Mild Intellectual Disabilities: Perspectives From Clinical Practice
Source: Front Psychol. 2020 Dec 10;11:572549. doi: 10.3389/fpsyg.2020.572549 (PMC7759728; doi:10.3389/fpsyg.2020.572549)
Supplement: Supplementary file 1 [file Data_Sheet_1.docx]

**APPENDIX A: Questioning Route**

|  | **Duration** | **What** | **Content / main questions** | **Probes** | **Result** | **Materials / conditions** |
| --- | --- | --- | --- | --- | --- | --- |
| 1 | 00.00-00.05 | **Introduction of the study** | Short introduction of the present study |  |  | Short PowerPoint presentation to explain research purposes/ Informed-consent forms. |
|  |  |  | Explaining the basic principles of the focus group meeting |  |  |  |
|  |  |  | Signing the inform-consent forms |  |  |  |
| 2 | 00.05-00.15 | **Introductory question** | Getting to know each other | *Where do you work?* | Breaking the ice.  Creating a safe and open atmosphere | One minute per participant (turn change by throwing a small ball?) |
|  |  |  |  | *Can you tell something about your work experience?* |  |  |

| 3 | 00.15-00:55 | **Research question 1** | Which interventions *within* the music do you use to lower tension or stress in patients with MID? | *What role do the different musical components play?* | Creating clear and concrete descriptions of interventions *within* the music  Categorization of interventions with respect to related goals, techniques, used instruments, and effects. | Moderator leads the discussion  Co-moderator makes notes |
| --- | --- | --- | --- | --- | --- | --- |
|  |  |  |  | *What is the duration of this specific intervention/exercise?* |  |  |
|  |  |  |  | *Can you make that a little bit more specific?* |  |  |
|  |  |  |  | *Can you give an example of that?* |  |  |
|  |  |  |  | *Would you call the intervention a music therapy technique?* |  |  |
|  |  |  |  | *How do you explain the effect of this intervention?* |  |  |

| 4 | 00.55-01.15 | **Research question 2** | Which intervention *outside* of the music do you use to lower tension or stress in patients with MID? | *What is the duration of this intervention/exercise?* | Clear and concrete descriptions of interventions outside of the music | Moderator leads the discussion  Co-moderator makes notes |
| --- | --- | --- | --- | --- | --- | --- |
|  |  |  |  | *Can you make that a little bit more specific?* |  |  |
|  |  |  |  | *How do you explain the effect of this intervention?* |  |  |
|  |  |  |  | *Can you give an example?* |  |  |
|  |  |  |  | *What aspects of the therapeutic attitude are important?* |  |  |

| 4 | 01.15-01.25 | **Research question 3** | Are there any factors that could influence the result or effect of the mentioned interventions? | *Can you make that a little bit more specific?* | Clear and concrete descriptions of effect-influencing factors | Moderator leads the discussion  Co-moderator makes notes |
| --- | --- | --- | --- | --- | --- | --- |
|  |  |  |  | *Why do you think this is an important factor?* |  |  |

| 5 | 01.25-01.30 | **Closing** | Are there any important questions we did not address, or additional comments? |  | Thanking the participants  Mention the member check | Thank-you gifts |
| --- | --- | --- | --- | --- | --- | --- |
|  |  |  | Thank everyone for their participations and hand out thank-you gifts |  |  | Checking the email addresses of participants |
|  |  |  | Indicate that a member check will take place after data analysis. |  |  |  |

APPENDIX B: Music Therapy Intervention Index

This index provides a more detailed description of the music therapy interventions mentioned by the music therapists of this study. Some interventions were only briefly mentioned, in which case we provided additional literature to explain the interventions.

| **Music therapy intervention** | **Description by the music therapists** | **Additional literature** |
| --- | --- | --- |
| Free improvisation | Free improvisation is mentioned across all three focus groups and described as a form of musical improvisation where no rules, restrictions or guidelines are imposed. The first step is to synchronize with the patient within the free improvisation. After reaching synchronization the music therapist can choose to change certain musical components to stimulate stress release or relaxation. | Improvisation and its various therapeutic uses and forms have been thoroughly described by Tony Wigram (2014) and Kenneth Bruscia (1987). One method worth mentioning is the Free Improvisation Therapy by Juliette Alvin (1975, 1976, 1978). |
| Freestyling | This intervention allows the patient to release tension by directly expressing their anger, fear and/or stress in music. The music therapist facilitates and guides this process. The patient chooses the type of beat, the instruments, etc.  “*This can be very difficult for patients with an intellectual disability … when they are relaxed, they are capable of verbalizing their story in a great way … but when they just have had bad news for example, a whole bucketload of frustration tends to come out*”. | Freestyle singing and rapping is used in music therapy settings to promote well-being through the expression and modulation of emotions, both positive and negative (Hadley & Yancy, 2012; Uhlig, Dimitriadis, Hakvoort, & Scherder, 2017). |
| 6/8-meter improvisation | The music therapist and patient(s) play a continuous 6/8 rhythm at a pulse of approximately 80 bpm. Variations in tempo and dynamics are limited as much as possible. This is sustained for approximately 10 minutes. This intervention can be experienced as a very meditative and relaxing exercise.  “*But at the same time, you turn inwards and come into contact with your emotions, some of which you might not want to be confronted with”*. | Wigram (2004) describes the use of pulsed improvisation.  Juslin, Liljeström, Västfjäll and Lundqvist (2010) describe the effect of rhythm and tempo on the emotional experience of music listening. |
| Interplay on piano | This intervention can be regarded as the beginning of an improvisation with the purpose to synchronize with the patient. The patient is asked to play their natural/intrinsic tempo on a single piano key. The music therapist accompanies their patient on the same piano, and the patient can be asked to choose between a major or minor key.  “*This allows me to get a feel for the patient’s state of mind at that moment*”. | Wigram (2014) describes how to execute a one-note improvisation and the significance of pulse in musical improvisation.  Juslin, Liljeström, Västfjäll and Lundqvist (2010) describe the process of *rhythmic entrainment*, where emotion is induced when the external rhythm of the music interacts with an internal body rhythm. |
| Expressing feelings in music | By using the musical components in a specific way, the patient tries to match the improvised music with their own feelings. This could be a way to express any negative emotions they are experiencing. | Symbolizing is an improvisational technique described by Bruscia (1987). It is the process of expressing personal feelings and experiences in music, to explore and modulate said feelings. |
| Taking turns | - | Taking turns (or turn-taking) refers to musical interactions in which the music therapist and patient take turns producing musical sounds. This reciprocal form of musical interaction depends on the fact that the patient can focus his/her social attention on his/her musical partner (Holck, 2002). |
| Mantra singing | Starting with just the voice, the music therapist sings mantras for or with the patient. The mantras can consist of just sounds, but a name can also be used as a mantra.  “*Many patients have never sung their own name, but it’s the most intrinsic thing you can do*”.  Looking to the pictures of loved ones while singing their names, can be a variation on this intervention. If needed, instrumental accompaniment van be added too. | The use of (improvised) vocal expressions to experience and express repressed emotions is a technique used in the music therapy method of therapeutic voice work (Austin, 2008; Baker & Uhlig, 2011). |
| Playing intrinsic tempo | This intervention uses the patient’s intrinsic tempo as the basis for a group music improvisation. The intervention begins with every group member playing their intrinsic tempo on a drum, all at the same time.  “*And so you get complete chaos, or at least you should*”.  Then, the music therapist implements structure into the improvisation. Once the improvisation has been structured, patients are asked to play their rhythm and the rest of the group mimics that rhythm. The patient is not only becoming aware of their intrinsic rhythm, but also how it feels to accommodate someone else’s rhythm. | Wigram (2004) describes the use and importance of pulsed improvisation.  Juslin, Liljeström, Västfjäll and Lundqvist (2010) describe the process of *Rhythmic entrainment*, where emotion is induced when the external rhythm of the music interacts with an internal body rhythm. |
| Greek Sirtaki | The music therapist plays a Greek Sirtaki on the accordion and patients play along with the rhythm on various percussion instruments. The pace is slowly increased until it culminates in shouting: “1.., 2.., 3.., 4.., HOPPA!”. During playing the Greek Sirtaki the music therapist can increase or decrease the music tempo. | The use of pre-existing music provides a set musical framework which gives the patient a sense of predictability and structure (Baker & Wigram, 2005) |
| Singing/Playing well-known songs | Well-known songs, such as children’s songs (nursery rhymes), folk songs and famous pop songs, are recognizable and provide a sense of structure. The musical preference of the patient and the symbolical meaning of a song can also come into play. | The lyrics and musical style of a well-known songs can have symbolic meaning to a patient (Aldridge, 2005; Austin, 2008; Baker & Uhlig, 2011).  The use of pre-existing music also provides a set musical framework which gives the patient a sense of predictability and structure (Baker & Wigram, 2005). |
| Keeping opening song constant | A short song which the therapist sings with or for the patient at the beginning of every music therapy session. This can be a pre-existing song, or a composed song by the music therapist.  “*It’s a way of creating rituals, which adds to the amount of structure and predictability”.* | - |
| Recording self-composed songs (and taking home) | Recordings of self-composed songs established during the music therapy sessions which can be taken home by the patient. The patient can use the recorded songs as relaxation material outside of the music therapy session. | The recording of composed songs can be part of the songwriting process (Baker & Wigram, 2005). |
| Songwriting | The method of songwriting was mentioned in combination with various goals. Songwriting can be used for short term stress release, by way of verbalizing frustrations, anger and fear within the music. However, it can also be offered as a form of exposure therapy for patients who suffer from long term stress as a result of difficult or traumatic experiences.  “*The more you work on a song, the less triggering the difficult experience becomes”.* | Songwriting as a music therapy method has various forms and goals, but is in essence the process of creating, practicing, performing and recording a song with the patient(s), during which the music therapist acts as a facilitator. (Baker & Wigram, 2005; Baker, 2015; Aasgaard and Ærø, 2016) |
| Hegi | Hegi’s Method of Musical Components is mentioned as a theoretic framework by several music therapists. | Hegi (1988, 1998) developed the Music Therapy Method of Musical Components, based on principles of the Gestalt therapy. Hegi describes the diagnostic and therapeutic value of various musical components, such as tone, rhythm, melody, dynamics and form. |
| Schumacher | A multi-sensory approach in which music is combined with other sensory inputs, such as visualization on a screen, body movement, or the vibration of an instrument, to activate multiple senses at the same time and subsequently achieve (internal) synchronization. | Karin Schumacher (1994, 2001) uses a combination of musical expression, visual contact, physical touch, and body movement, to emulate the early mother-child relationship to promote emotional development. |
| Ronnie Gardiner Method | The Ronnie Gardiner Method uses symbols instead of musical notes. Every symbol represents a movement and a word which are executed and said to the beat of the music. Through this manner various areas of the brain are stimulated at the same time. Since the exercise requires a lot of concentration | More information on the Ronnie Gardiner Method, as well as links to various publications, can be found on the website “Ronnie Gardiner Method” (2015). |
| NMT-MACT | - | The Musical Attention Control Training (MACT) is one of the interventions of the Neurologic Music Therapy (NMT). The MACT described by Thaut and Gardiner (2014) provides “structured active or perceptive musical exercises involving precomposed performance or improvisation in which musical elements cue different musical responses to practice attention functions.” The method focusses on three different aspects of attention control: focused attention (one task), sustained attention, and alternated attention. |
| One Note Symphony (Berman) | - | The One note symphony is a music therapy method by Albert Berman (2016). It is a group improvisation where patients are asked to play a single note on a broad range of instruments, creating a melody only through interaction and utilizing different rhythmic forms. With the help of this intervention, Berman tries to make group improvisation accessible to people who do not have complex musical skills and thus enable more interaction and connection between patients during musical improvisation. |
| Recording and/or creating personalized relaxation music | Personal playlists can be used by the patient outside of the music therapy session to achieve relaxation. These playlists can include pre-existing songs, recorded compositions by the patient and/or music therapist, and recorded improvisations made during the music therapy sessions. | Creating a personalized playlist for patients to induce relaxation is a form of receptive music therapy which can be used both during and outside of the music therapy session (Grocke & Wigram, 2007). |

**References Appendix B**

Aasgaard, T., & Ærø, S. B. (2016). Songwriting techniques in music therapy practice. In J. Edwards (Ed.), *The Oxford handbook of music therapy* (pp. 644-668). Oxford: Oxford University Press.

Aldridge, D. (Ed.). (2005). *Case study designs in music therapy.* London: Jessica Kingsley Publishers.

Alvin, J. (1975). *Music therapy* (revised edition). London: John Claire Books.

Alvin, J. (1976). *Music therapy for the handicapped child* (second edition). London: Oxford University Press.

Alvin, J. (1978). *Music therapy for the autistic child*. London: Oxford University Press.

Austin, D. (2008). *The theory and practice of vocal psychotherapy*. London: Jessica Kingsley Publishers.

Baker, F. (2015). *Therapeutic songwriting*. New York: Palgrave Macmillan.

Baker, F., & Uhlig, S. (Eds.). (2011). *Voicework in music therapy research and practice*. London: Jessica Kingsley Publishers.

Baker, F., & Wigram, T. (2005). *Songwriting: methods, techniques and clinical applications for music therapy clinicians, educators and students*. London: Jessica Kingsley Publishers.

Berman, A. (2016). One note symphony. *Nordic Journal of Music Therapy*, *25*, 99-99. https://doi.org/10.1080/08098131.2016.1180105

Bruscia, K. E. (1987). *Improvisational models of music therapy*. Springfield: Charles. C. Thomas.

Grocke, D., & Wigram, T. (2007). *Receptive methods in music Therapy: Techniques and clinical applications for music therapy clinicians, educators and students*. London: Jessica Kingsley Publishers.

Hadley, S., & Yancy, G. (Eds.). (2012). *Therapeutic uses of rap and hip hop*. New York: Rouledge.

Hegi, F. (1988). *Improvisation und Musiktherapie* [Improvisation and music therapy]. Paderborn: Junfermann Verlag.

Hegi, F. (1998). *Übergänge zwischen Sprache und Musik: Die Wirkungskomponenten der Musiktherapie* [Transitions between speaking and music: The working mechanisms of music therapy]. München/Basel: Ernst Reinhard Verlag.

Holck, U. (2002). Music Therapy for Children with Communication Disorders. In. T. Wigram, I. N. Pedersen, & L. O. Bonde (Eds.), *A comprehensive guide to music therapy: Theory, clinical practice, research and training* (pp. 183-187). London: Jessica Kingsley Publishers.

Juslin, P. N., Liljeström, S., Västfjäll, D., & Lundqvist, L. (2010). How does music evoke emotions? Exploring the underlying mechanisms. In P. N. Juslin & J. A. Sloboda (Eds.), *Handbook of music and emotion: Theory, research, applications* (pp. 605-642). Oxford: Oxford university Press.

Ronnie Gardiner Method: Start. (2015). Retrieved May 29, 2020, from RGM website http://www.ronniegardinermethod.com/

Schumacher, K. (1994). *Musiktherapie mit autistischen Kindern* [Music therapy with autistic children]. Stuttgart: Gastav Fischer Verlag.

Thaut, M. H., & Gardiner, J. C. (2014). Musical Attention Control Training. In M. H. Thaut, V. Hoemberg (Eds.), *Handbook of neurologic music therapy* (pp. 257-269). Oxford: Oxford University Press.

Uhlig, S., Dimitriadis, T., Hakvoort, L., & Scherder, E. (2017). Rap and singing are used by music therapists to enhance emotional self-regulation of youth: Results of a survey of music therapists in the Netherlands. *The Arts in Psychotherapy*, *53*, 44-54. <https://doi.org/10.1016/j.aip.2016.12.001>

Wigram, T. (2004). *Improvisation: Methods and techniques for music therapy clinicians, educators and students*. London: Jessica Kingsley Publishers.
